# Memory complaint scale (MCS). Proposed tool for active systematic search

**DOI:** 10.1590/S1980-57642012DN06040004

**Published:** 2012

**Authors:** Francisco A.C. Vale, Ari P. Balieiro-Jr, José Humberto Silva-Filho

**Affiliations:** 1 PhD, Adjunct Professor of Medicine of the Federal University of São Carlos (UFSCar), Neurologist, São Carlos SP, Brazil.; 2 Masters, Assistant Researcher of the Cognitive and Behavioral Neurology Group of the UFSCar and the Laboratory of Psychological Assessment of the UFAM, psychologist.; 3 PhD, Adjunct Professor of the School of Psychology of the Federal University of Amazonas (UFAM), psychologist, Manaus AM, Brazil.

**Keywords:** subjective memory complaints, memory, psychometric tests, dementia

## Abstract

Subjective Memory Complaints (SMC) are frequent among adults and elderly and are associated with poor quality of life. The etiology and clinical significance of SMCs are unclear, but these complaints are associated with objective cognitive decline or with depression, anxiety and psychosocial stressors. Biological and physiological brain alterations resembling those in Alzheimer's Disease have been found in SMC. SMC can evolve with different outcomes and represent the initial symptom or a risk factor of dementia. Active systematic search can be useful for early screening of candidates for preventive or therapeutic interventions.

**Objective:**

To propose a Memory Complaints Scale (MCS) as an instrument for actively searching for memory complaints and to investigate its utility for discriminating demented from cognitively normal elderly.

**Methods:**

A total of 161 patients from a teaching behavioral neurology outpatient unit of a tertiary hospital were studied. The MCS was used in two ways, by direct application to the patient and by application to the patient's companion. Cognitive tests assessing depression and daily living activities were also applied.

**Results:**

High Cronbach's alpha coefficients were found for the two application methods. Correlations between the two versions and the other instruments administered for patients grouped by type and severity of dementia were also found.

**Conclusion:**

The MCS is a useful scale for identifying memory complaints and discriminating demented from cognitively normal elderly. Further studies confirming these findings are warranted.

## Abstract (pt)

Queixa Subjetiva de Memória (QSM) é frequente entre adultos e idosos e está associada a pior qualidade de vida. Etiologia e significado clínico são incertos, sendo associada a perdas cognitivas objetivas ou a depressão, ansiedade e estressores psicossociais. Foram demonstradas alterações biológicas e fisiológicas encefálicas semelhantes às da doença de Alzheimer. Pode ter diferentes desfechos e representar sintoma inicial ou fator de risco para demência. A busca ativa e sistematizada pode ser útil na identificação precoce de pessoas que poderão receber intervenções preventivas ou terapêuticas.

**Objetivo:**

Propor a Escala de Queixa de Memória (EQM) como um instrumento para a busca de queixa de memória e investigar se é útil para discriminar idosos demenciados de normais.

**Métodos:**

Foram estudados 161 pacientes de um ambulatório didático de neurologia comportamental de um hospital terciário. A EQM foi utilizada nas duas formas, uma diretamente aplicada ao paciente e a outra aplicada ao acompanhante sobre o paciente. Também foram aplicados testes cognitivos, para depressão e para atividades diárias.

**Resultados:**

Foram encontrados altos coeficientes alfa de Cronbach para as duas formas. Também foram encontradas correlações entre as duas formas e os outros instrumentos, para os pacientes agrupados conforme tipo e gravidade da demência.

**Conclusão:**

A EQM é uma escala útil para identificar queixa de memória e pode ser útil para discriminar idosos demenciados de normais. Estudos subsequentes deverão ser realizados para verificar essas informações.

## INTRODUCTION

The term Subjective Memory Complaint (SMC) is used generally to designate a report of memory problems which may or may not be perceived by others, although there is currently no consensus on a standard definition for this symptom. Subjective Cognitive Complaint (SCC) and Subjective Memory Impairment (SMI) are other terms used to describe the same symptom.^1^

SMC is a frequent symptom among adults and elderly the prevalence of which increases with age. Population-based studies estimate prevalences as high as 46.3% in adults 50-59 years old and 63.4% in older old 80-100 years of age. Female gender and low educational level have also been associated with higher prevalences of SMC.^2-4^ Two Brazilian population samples with different cultural and sociodemographic characteristics, one located in the Northern and the other in the Southern region, estimated SMC prevalences at 70.0% and 56.0%, respectively.^5,6^

Data in the literature vary widely regarding the etiology and clinical significance of SMC, with studies reporting conflicting results. Studies involving population samples have shown that SMC is associated with impaired performance on memory tests, in elderly without dementia or depression^4,7^ and may predict dementia by up to three years, particularly if associated with objective memory deficits.^8^ Other studies however, have associated SMC with psychosocial stress, anxiety or depression.^9,10^

Mild Cognitive Impairment (MCI) includes SMC as a key diagnostic criterion.^11^ There is evidence suggesting that SMC in elderly is a significant risk factor for MCI ^12^ or for dementia.^13,14^

Some studies have shown biological or physiological brain changes in SMC which closely resemble those seen in Alzheimer's Disease (AD), based on structural, functional and metabolic neuroimaging,^15-18^ as well as electroencephalographic^19^, magnetoencephalographic,^20^ genetic,^21^ and neuropathologic studies.^22^ A recent study has shown that hippocampal volumes correlate with outcomes of memory training interventions in adults with MCI.^23^

In another recent study, cognitive decline was evident in subjects from 45 years of age and older.^24^ However, even when individuals report symptoms and exhibit objective deficit, dementia may not be diagnosed. Up to 75% of patients with moderate to severe dementia may not be identified by the General Practitioner as having cognitive disorders while up to 97% of patients with mild cognitive disorders are not identified as having incipient dementia.^25^

SMCs in the elderly are associated with poorer quality of life and impaired activities of daily living (ADL)^26^ and generate costs with the utilization of public primary health care services.^27^

Particularly among the elderly, SMC should not be attributed to a harmless phenomenon of senescence or a symptom or depression. The condition is polymorphic with different outcomes and may represent an initial symptom of dementia or a risk factor for future dementia. Therefore, SMC should be taken seriously warranting a thorough investigation and follow-up.^4,28^

Active systematic search can be useful for early screening of at-risk individuals with SMC, enabling prompt preventive or therapeutic interventions.

The aim of this study was to propose a structured questionnaire (Memory Complaints Scale - MCS) as an instrument for actively searching for memory complaints, and to investigate Its utility for discriminating demented from cognitively normal elderly.

## METHODS

**Casuistic.** The study data were collected directly from patients aged 60 years and older and also from their companions, at the Behavioral Neurology Outpatient Unit of the Clínicas Hospital of the Ribeirão Preto School of Medicine of the University of São Paulo (ANCP-HCFMRP) over a period spanning 18 months. The sample comprised 161 subjects, 59.0% of female gender. Mean age was 72.0±7.67 years and mean schooling was 4.6±3.2 years. Of the participants, 5.0% were single, 60.2% married, 3.1% separated and 31.7% widowed. After full clinical and laboratory assessments, 28.0% of patients were diagnosed with AD, 26.7% MCI, 16.8% vascular dementia, 26.1% other dementia types and 2.5% with SMC.

**Instruments.** Memory Complaint Scale (MCS). MCS (Appendices 1 and 2) has been used as part of the routine protocols of two teaching outpatient clinics, previously by the ANCP-HCFMRP^29^ and currently by the Interdisciplinary Outpatient Unit of Neurology of the UFSCar (ANEU-UFSCar).^30^ The MCS is a scale designed for carrying out a systematic active search for memory complaints. It comprises a questionnaire containing seven questions with graded responses of increasing intensity (0, 1 and 2). The test subject is classified in terms of memory complaint (MC) based on their score as follows: No MC (0-2), mild MC (3-6), moderate MC (7-10) or severe MC (11-14). The Scale has two versions, one for application directly to the test subject (MCS-A) and another for application to the companion (MCS-B). Both versions contain the same items, but the first is a self-report version while in the second the companion describes their observations concerning the patient's memory. The instrument explores the frequency of complaints and the degree these problems impact everyday activities, and also seeks to compare current memory with that at a younger age and with the memory of others of similar age. Both versions were employed in this study.

Other assessment instruments included in the cited protocols were: Mini-Mental State Exam (MMSE);^31,32^ Clinical Dementia Rating (CDR);^33,34^ Words List (immediate recall, delayed recall and recognition) adapted from the CERAD;^35^ Clock Drawing Test;^36^ Geriatric Depression Scale (GDS);^37,38^ Pfeffer Functional Activities Questionnaire (FAQ);^39^ and Frontal Assessment Battery.^40,41^

**Procedure.** This study was conducted at the ANCP-HCFMRP and approved by the Research Ethics Committee of the HCFMRP-USP (Under CAAE 0387.0.004.000-07). This was a correlational prospective correlational study involving a randomly selected sample drawn from the casuistic of a specialized outpatient unit of a teaching hospital. The data were collected on an individual basis through two visits with the elderly and their companion, specifically assessing the clinical, cognitive and functional status of the patient. Data were analyzed in an effort to initially check the validity and reliability of the MCS-A and MCS-B using Cronbach's alpha, while also investigating the item-total correlation. Subsequently, the data obtained using the two versions of the scale were stratified into four subgroups by CDR (0, 1, 2 and 3) in order to assess the informative and discriminative power of the two MCSs (A and B), comparing the results on the scales against the results found on the MMSE. The data found in these four groups were submitted to Multivariate Analysis (ANOVA) in order to identify any statistically significant differences among them. Finally, in order to explore the informative and predictive power of the MCS instruments, correlation studies were performed between the scores obtained using versions A and B, and the results on cognitive tests from the protocol of the outpatient unit, specifically on the previously mentioned tests.

## RESULTS

**Internal consistency of the MCS-A and MCS-B.** With regard to the MCS-A (self-report), a high Alpha coefficient (0.850) was found along with item-total correlations greater than 0.512 on the seven items of the scale. With the regard to the MCS-B (companion report), a similarly high Alpha coefficient (0.847) was found and item-total correlations greater than 0.470. The coefficients found for both scales proved reliable (above 0.080) indicating good internal consistency of the data. Correlations of the items with total score of each scale were all greater than 0.30, indicating that all items had good informative properties for the construct investigated, with no need or desire to remove any of the items from either scale for adjustment purposes.

**Analysis of subgroups by CDR.** The sample was stratified into four subgroups by CDR (0, 1, 2 and 3) in order to assess the informative and discriminative potential of the MCS-A and MCS-B, comparing the results on the scales against mean values on the MMSE for each subgroup. The results shown in Table 1, indicate that the MCS-A (self-report) had higher memory complaint scores in milder clinical conditions (CDR 0 and 1) and less intense scores in more advanced clinical conditions (CDR 2 and 3). Moreover, comparison of the patient self-report (MCS-A) in the first subgroup (CDR=0) revealed that in this category, indicating absence of dementia, the mean memory complaint score was 7.40, higher than the mean score on the MCS-B (companion report) of 5.58. These results appear to show that, although not recognized by the companion, a memory problem was already perceived by the patients even in the absence of a dementia condition.

**Table 1:** Subgroups by CDR.

CDR	Indicators	Mean	SD
0 (N=43)	MCS-A	7.40	4.204
	MCS-B	5.58	5.225
	MMSE	23.20	4.468
1 (N=50)	MCS-A	7.74	4.075
	MCS-B	9.54	4.372
	MMSE	17.78	4.129
2 (N=34)	MCS-A	5.15	4.009
	MCS-B	11.26	3.848
	MMSE	14.78	4.145
3 (N=23)	MCS-A	4.96	3.948
	MCS-B	12.09	2.859
	MMSE	7.93	6.070

CDR: Clinical Dementia Rating; SD: standard deviation.

Results showed that, on average, patients with CDR 1 reported an MC closer to CDR 0, whereas the reported intensity of their complaint reduced progressively at CDR 2 and 3, suggesting the occurrence of anosognosia, a common symptom in dementia conditions. On the MCS-B however, a growing number of MCs were reported accompanying the progression in the dementia condition. The same trend was evident for MMSE scores in each subgroup, with decreasing scores as dementia progressed. Multivariate analysis (ANOVA) comparing the means for the MCS-A, MCS-B and MMSE among the four CDR subgroups (0, 1, 2 and 3), confirmed statistically significant differences between means on the MCS-A (p≤0.05), and likewise for the MCS-B and MMSE in each group (p≤0.01).

Significant correlations with other instruments: Studying the overall sample in search of correlations between scores on the MCS A and B and the other assessment instruments revealed various significant correlations, albeit of weak to reasonable intensities. Most notable however, were the correlations between scores obtained on the MCS-B and the Pfeffer Functional Assessment Questionnaire (0.470, p< 0.01), and between the MCS-B and the CDR (0.509, p< 0.01) (Table 2).

**Table 2:** Significant Correlations of MCS-A + B with other instruments.

	MCS-A	MCS-B
Age (N=161)	-0.219**	-
MMSE (N=113)	0.241*	-0.321**
CDR (N=150)	-0.246**	0.470**
Words list (Immediate recall) (N=157)	0.241**	-0.330**
Words list (Delayed recall) (N=154)	0.240**	-0.325**
Words list (Recognition) (N=146)	-	-0.272**
Clock Drawing Test (N=137)	0.304**	-0.246**
Functional Assessment Questionnaire (N=161)	-	0.509**
Frontal Assessment Battery (N=161)	0.247**	-0.250**
Geriatric Depression Scale (N=144)	0.374**	-

* p<0.05;

** p<0.01.

Subgroups of the overall sample were also explored to identify correlations. In the subgroup containing patients diagnosed with AD and those with cerebral vascular disease, correlations were identified between scores on the MCS-B and on the Pfeffer-FAQ (0.383, p< 0.01); as well as on the CDR (0.407, p< 0.01). In the subgroup formed by only patients with AD diagnosis, correlations of 0.497 (p< 0.01) between the MCS-B and Pfeffer-FAQ; and of 0.512 (p< 0.01) between the MCS-B and CDR, were detected.

Table 2 highlights the statistically significant weak positive correlations between the MCS-A and performance on cognitive tests, in addition to a positive correlation (reasonable to good) with depression, suggesting that cognitively functional individuals seeking neurological assistance may have MC which is possibly associated to other psychic problems.

At the same time, statistically significant inverse correlations were seen (weak to reasonable) between MCS-B and performance on cognitive tests. These results suggest that the higher the MC reported by the companion the lower the performance by the patient on cognitive tests. In addition, a weak inverse correlation was also observed between MCS-A and age, i.e. in this sample, the older individuals tended to exhibit fewer MCs.

## DISCUSSION

A number of different types of validated questionnaires are available for assessing SMC^3,6,13,42-45^ but are extensive or fail to effectively discriminate SMC from dementia.

A Memory Complaint Scale (MCS) was proposed in the present study. It was decided to designate the scale a Memory Complaint (MC) instrument because a subjective memory complaint, as commonly used in the literature, is redundant in the sense that all complaints by definition refer to a subjective symptom.

The results of this study showed that the MCS is a stable, informative and discriminate scale, for both versions A and B. These results corroborate previous reports validating the scale.^46-48^

Data given in Table 1 shows that elderly without dementia can complain of memory problems even though the companion does not recognize them. However, patients with mild dementia reported MCs in a similar manner to those without dementia, where the intensity of complaints reduced progressively with advancing dementia, probably due to anosognosia, a frequent symptom in dementia conditions.^49^ Conversely, reports by the companion increased progressively with advancing dementia. The same phenomenon was observed regarding MMSE scores, with progressively lower scores accompanying the evolution of the dementia.

In patients with AD, reports by the companion correlated with patient performance on ADLs and severity of dementia. In preliminary results reported previously, the MCS was considered a useful tool since although anosognosic patients self-assessed as having no dementia, the discrepancy with the assessment by the companions is itself discriminative. The same holds true for patients with dementia in general.^46,48^

The data contained in Table 2 shows the weak positive correlations between patient-reported MCs and performance on tests of memory and executive functions. The results also evidence a positive correlation (reasonable to good) with the depressive symptoms questioned, suggesting that cognitively functional individuals seeking neurological assistance may have MCs which could be associated to depression. Other studies in outpatient casuistics have also shown an association between MCs and depression, as well as with anxiety and psychosocial stressors.^9,10^ On the other hand, MCs are common among adults and often a source of stress and concern.^50^

These findings also showed negative correlations (weak to reasonable) between patient memory problems as reported by the companion and performance on tests of memory, executive functions and CDR, suggesting that the worse the patient's cognitive performance, the more intense the report by the companion. The same pattern was seen for patient performance on activities of daily living. Other studies have affirmed that MCs are associated with performance on memory tests, even after controlling for number of depressive symptoms.^4,7^ In addition, a weak negative correlation was also observed between MCS-A and age, suggesting that in this sample of patients from a specialized outpatient clinic, older individuals tended to exhibit fewer MCs. However, population-based studies suggest that age is generally associated with MCs, independently of degree of cognitive functioning.^3,4^

Based on these results, it can be concluded that the MCS, used in its two versions, is a useful scale for active systematic and consistent search for memory complaints, and may be used to discriminate demented from cognitively normal elderly. Further studies to confirm these findings are warranted.

## APPENDIX

**Table:**

**MCS - MEMORY COMPLAINT SCALE**		
VERSION A - PATIENT ANSWERS		
Objective: To assess patient's memory complaint directly with him/her		
**Instructions:**	• Apply this directly to patient with no intervention from companion • Read aloud in a clear voice	
**Q1. Do you have any memory problems? (or "forgetfulness?" or "memory difficulties") **		
( ) No = 0	( ) Unable to answer/unsure/doubt = 1	( ) Yes = 2
If answers No, mark 0 and likewise for Q2 and Q3 and skip ahead to Q4		
**Q2. How often does this happen?**		
( ) Rarely = 0	( ) Occasionally/sometimes =1	( ) A lot/frequently = 2
**Q3. Does this memory problem hamper (or impair) your daily activities?**		
( ) No = 0	( ) Occasionally/sometimes = 1	( ) A lot /frequently = 2
**Q4. How is your memory compared to others your age?**		
( ) The same or better = 0	( ) Somewhat worse = 1	( ) Much worse = 2
**Q5. How is your memory compared with when you were younger?**		
( ) Same or better = 0	( ) Somewhat worse = 1	( ) Much worse = 2
**Q6. Do you forget what you've just read or heard (e.g., in a conversation)?**		
( ) Rarely/never = 0	( ) Occasionally = 1	( ) Often = 2
**Q7. Rate your memory on a scale of 1 to 10, with 1 worst and 10 best**		
( ) 9 or 10 = 0	( ) 5 to 8 = 1	( ) 1 to 4 = 2
Scoring		
**Interpretation**		
[ ] No MCs (0-2) [ ] Mild MCs (3-6) [ ] Moderate MCs (7-10) [ ] Severe MCs (11-14)		
		
**MCS - MEMORY COMPLAINT SCALE**		
VERSION B - COMPANION ANSWERS ABOUT PATIENT		
**Objective:** To assess memory complaint of patient by companion report		
**Instructions:**	• Apply with the companion referring to patient • Read aloud in clear voice	
**Q1. Does he/she have a memory problem ? (or "forgetfulness?")**		
( ) No = 0	( ) Unable to answer/unsure/doubt = 1	( ) Yes = 2
If answers No, mark 0 and likewise for Q2 and Q3 and skip ahead to Q4		
**Q2. How often does this happen?**		
( ) Rarely = 0	( ) Occasionally/sometimes =1	( ) A lot /frequently= 2
**Q3. Does this memory problem hamper (or impair) his/her daily activities?**		
( ) No = 0	( ) Occasionally/sometimes = 1	( ) A lot /frequently = 2
**Q4. How is his/her memory compared to others their age?**		
( ) The same or better = 0	( ) Somewhat worse = 1	( ) Much worse = 2
**Q5. How is his/her memory compared with when they were younger?**		
( ) The same or better = 0	( ) Somewhat worse = 1	( ) Much worse = 2
**Q6. Does he/she forget what they've just read or heard (e.g., in a conversation)?**		
( ) Rarely/never = 0	( ) Occasionally = 1	( ) Often = 2
**Q7. Rate his/her memory on a scale of 1 to 10, with 1 worst and 10 best**		
( ) 9 or 10 = 0	( ) 5 to 8 = 1	( ) 1 to 4 = 2
Scoring		
**Interpretation**		
[ ] No MCs (0-2) [ ] Mild MCs (3-6) [ ] Moderate MCs (7-10) [ ] Severe MCs (11-14)		
**The Portuguese version of the Memory Complaint Scale is available at: www.demneuropsy.com.br**		

**Table:** EQM - ESCALA DE QUEIXA DE MEMÓRIA FORMA A + PACIENTE RESPONDE

Objetivo: Avaliar a queixa de memória do(a) paciente, diretamente com ele(a)		

**Instruções**		
• Aplique diretamente com o(a) paciente, sem a intervenção do(a) acompanhante		
• Leia em voz alta e clara		

P1. Você tem problema de memória? (**ou** “de esquecimento?" **ou** “dificuldade de memória”)		
( ) Não = 0	( ) Não sabe responder/indeciso/dúvida = 1	( ) Sim = 2
Se responder **Não**, marque 0 também na P2 e na P3 e pule para a P4		

P2. Com que frequência esse problema acontece?		
( ) Raramente = 0	( ) Pouco/mais ou menos =1	( ) Muito/frequente = 2

P3. Esse problema de memória tem atrapalhado (**ou** prejudicado) suas atividades no dia-a-dia?		
( ) Não = 0	( ) Pouco/mais ou menos = 1	( ) Muito/frequente = 2

P4. Como está sua memória em comparação com a de outras pessoas de sua idade?		
( ) Igual ou melhor = 0	( ) Um pouco pior = 1	( ) Muito pior = 2

P5. Como está sua memória em comparação a quando você era mais jovem?		
( ) Igual ou melhor = 0	( ) Um pouco pior = 1	( ) Bem pior = 2

P6. Acontece de você esquecer o que acabou de ler ou de ouvir (p. ex., numa conversa)?		
( ) Raramente/nunca = 0	( ) De vez em quando = 1	( ) Frequentemente = 2

P7. Dê uma nota de 1 a 10 para sua memória, sendo 1 a pior e 10 a melhor.		
( ) 9 ou 10 = 0	( ) 5 a 8 = 1	( ) 1 a 4 = 2

**Pontuação ______**		

**Interpretação:**		
[ ] Sem QM (0-2) [ ] QM leve (3-6) [ ] QM moderada (7-10) [ ] QM acentuada (11-14)		

**Table:** EQM - ESCALA DE QUEIXA DE MEMÓRIA FORMA B + ACOMPANHANTE RESPONDE SOBRE PACIENTE

Objetivo: Avaliar a queixa de memória do(a) paciente por intermédio do(a) acompanhante		

**Instruções**		
• Aplique com o acompanhante referindo-se à(o) paciente.		
• Leia em voz alta e clara		

P1. Ele(a) tem problema de memória? (**ou** "de esquecimento?")		
( ) Não = 0	( ) Não sabe responder/indeciso/dúvida = 1	( ) Sim = 2
Se responder **Não**, marque 0 também na P2 e na P3 e pule para a P4		

P2. Com que frequência esse problema acontece?		
( ) Raramente = 0	( ) Pouco/mais ou menos =1	( ) Muito/frequente = 2

P3. Esse problema de memória tem atrapalhado (ou prejudicado) atividades dele(a) no dia-a-dia?		
( ) Não = 0	( ) Pouco/mais ou menos = 1	( ) Muito/frequente = 2

P4. Como está a memória dele(a) em comparação com a de outras pessoas de sua idade?		
( ) Igual ou melhor = 0	( ) Um pouco pior = 1	( ) Muito pior = 2

P5. Como está a memória dele(a) em comparação a quando era mais jovem?		
( ) Igual ou melhor = 0	( ) Um pouco pior = 1	( ) Bem pior = 2

P6. Acontece de ele(a) esquecer o que acabou de ler ou de ouvir (p. ex., numa conversa)?		
( ) Raramente/nunca = 0	( ) De vez em quando = 1	( ) Frequentemente = 2

P7. Dê uma nota de 1 a 10 para a memória dele(a), sendo 1 a pior e 10 a melhor.		
( ) 9 ou 10 = 0	( ) 5 a 8 = 1	( ) 1 a 4 = 2

**Pontuação ______**		

**Interpretação:**		
[ ] Sem QM (0-2) [ ] QM leve (3-6) [ ] QM moderada (7-10) [ ] QM acentuada (11-14)		

## References

[1] Abdulrab K, Heun R (2008). Subjective Memory Impairment. A review of its definitions
indicates the need for a comprehensive set of standardised and validated
criteria. Euro Psychiatry.

[2] Westoby CJ, Mallen CD, Thomas E (2009). Cognitive complaints in a general population of older adults:
prevalence, association with pain and the influence of concurrent affective
disorders. Eur J Pain.

[3] Park MH, Min JY, Min HY, Lee HJ, Lee DH, Song MS (2007). Subjective memory complaints and clinical characteristics in
elderly Koreans: a questionnaire survey. Int J Nurs Stud.

[4] Jonker C, Geerlings MI, Schmand B (2000). Are memory complaints predictive for dementia? A review of
clinical and population-based studies. Int J Geriatr Psychiatry.

[5] Brucki SM, Nitrini R (2009). Subjective memory impairment in a rural population with low
education in the Amazon rainforest: an exploratory study. Int Psychogeriatr.

[6] Xavier F, Ferraz MPT, Argimon I, Moriguchi EH (2001). The prevalence of a subjective perception of loss of memory in
the elderly. Rev Bras Neurol.

[7] Snitz BE, Morrow LA, Rodriguez EG, Huber KA, Saxton JA (2008). Subjective memory complaints and concurrent memory performance in
older patients of primary care providers. J Int Neuropsychol Soc.

[8] Schmand B, Jonker C, Geerlings MI, Lindeboom J (1997). Subjective memory complaints in the elderly: depressive symptoms
and future dementia. Br J Psychiatry.

[9] Elfgren C, Gustafson L, Vestberg S, Passant U (2010). Subjective memory complaints, neuropsychological performance and
psychiatric variables in memory clinic attendees: a 3-year follow-up
study. Arch Gerontol Geriatr.

[10] Fischer CE, Jiang D, Schweizer TA (2010). Determining the association of medical co-morbidity with
subjective and objective cognitive performance in an inner city memory
disorders clinic: a retrospective chart review. BMC Geriatr.

[11] Petersen RC, Stevens JC, Ganguli M, Tangalos EG, Cummings JL, DeKosky ST (2001). Practice parameter: early detection of dementia: mild cognitive
impairment (an evidence-based review). Report of the Quality Standards
Subcommittee of the American Academy of Neurology. Neurology.

[12] Luck T, Riedel-Heller SG, Luppa M (2010). Risk factors for incident mild cognitive impairment--results from
the German Study on Ageing, Cognition and Dementia in Primary Care Patients
(AgeCoDe). Acta Psychiatr Scand.

[13] Schmand B, Jonker C, Hooijer C, Lindeboom J (1996). Subjective memory complaints may announce
dementia. Neurology.

[14] Wang L, Belle Gv, Crane PK (2004). Subjective Memory Deterioration and Future Dementia in People
Aged 65 and Older. J Am Geriatr Soc.

[15] Stewart R, Dufouil C, Godin O (2008). Neuroimaging correlates of subjective memory deficits in a
community population. Neurology.

[16] Jessen F, Feyen L, Freymann K (2006). Volume reduction of the entorhinal cortex in subjective memory
impairment. Neurobiol Aging.

[17] Rodda J, Okello A, Edison P, Dannhauser T, Brooks DJ (2010). (11)C-PIB PET in subjective cognitive impairment. Eur Psychiatry.

[18] Hohman TJ, Beason-Held LL, Lamar M, Resnick SM (2011). Subjective cognitive complaints and longitudinal changes in
memory and brain function. Neuropsychology.

[19] Babiloni C, Visser PJ, Frisoni G (2010). Cortical sources of resting EEG rhythms in mild cognitive
impairment and subjective memory complaint. Neurobiol Aging.

[20] Maestu F, Baykova E, Ruiz JM (2011). Increased biomagnetic activity in healthy elderly with subjective
memory complaints. Clin Neurophysiol.

[21] Dik MG, Jonker C, Comijs HC (2001). Memory complaints and APOE- 4 accelerate cognitive decline in
cognitively normal elderly. Neurology.

[22] Barnes LL, Schneider JA, Boyle PA, Bienias JL, Bennett DA (2006). Memory complaints are related to Alzheimer disease pathology in
older persons. Neurology.

[23] Engvig A, Fjell AM, Westlye LT, Skaane NV, Sundseth Ø, Walhovd KB (2012). Hippocampal subfield volumes correlate with memory training
benefit in subjective memory impairment. NeuroImage.

[24] Singh-Manoux A, Kivimaki M, Glymour MM (2012). Timing of onset of cognitive decline: results from Whitehall II
prospective cohort study. BMJ.

[25] Gifford DR, Cummings JL (1999). Evaluating dementia screening tests: methodologic standards to
rate their performance. Neurology.

[26] Montejo P, Montenegro M, Fernández MA, Maestú F (2012). Memory complaints in the elderly: Quality of life and daily
living activities. A population based study. Arch Gerontol Geriatr.

[27] Waldorff F, Siersma V, Waldemar G (2009). Association between subjective memory complaints and health care
utilisation: a three-year follow up. BMC Geriatrics.

[28] Gallassi R, Oppi F, Poda R (2010). Are subjective cognitive complaints a risk factor for
dementia. Neurol Sci.

[29] Vale FAC, Balieiro-Júnior AP, Silva-Filho JH (2006). Manual de Procedimentos de Rotina - Revisado (MPR-Rev) do
Ambulatório de Neurologia Comportamental do Hospital das
Clínicas da Faculdade de Medicina de Ribeirão Preto da
Universidade de São Paulo.

[30] Vale FAC (2011). Manual de Procedimentos de Rotina do Ambulatório Interdisciplinar
de Neurologia da Universidade Federal de São Carlos
(ANEUUFSCar).

[31] Folstein MF, Folstein SE, McHugh PR (1975). "Mini-mental state". A practical method for grading the cognitive
state of patients for the clinician. J Psychiatr Res.

[32] Brucki SM, Nitrini R, Caramelli P, Bertolucci PH, Okamoto IH (2003). Suggestions for utilization of the mini-mental state examination
in Brazil. Arq Neuropsiquiatr.

[33] Hughes CP, Berg L, Danziger WL, Coben LA, Martin RL (1982). A new clinical scale for the staging of dementia. Br J Psychiatry.

[34] Chaves ML, Camozzato AL, Godinho C (2007). Validity of the clinical dementia rating scale for the detection
and staging of dementia in Brazilian patients. Alzheimer Dis Assoc Disord.

[35] Bertolucci P, Okamoto I, Brucki S, Siviero M, Toniolo-Neto J, Ramos L (2001). Applicability of the CERAD neuropsychological battery to
Brazilian elderly. Arq Neuropsiquiatr.

[36] Sunderland T, Hill JL, Mellow AM (1989). Clock drawing in Alzheimer's disease. A novel measure of dementia
severity. J Am Geriatr Soc.

[37] Yesavage JA, Brink TL, Rose TL (1983). Development and validation of a geriatric depression screening
scale: a preliminary report. J Psychiatr Res.

[38] Almeida OP, Almeida SA (1999). Short versions of the geriatric depression scale: a study of
their validity for the diagnosis of a major depressive episode according to
ICD-10 and DSM-IV. Int J Geriatr Psychiatry.

[39] Pfeffer RI, Kurosaki TT, Harrah CH Jr., Chance JM, Filos S (1982). Measurement of functional activities in older adults in the
community. J Gerontol.

[40] Beato RG, Nitrini R, Formigoni AP, Caramelli P (2007). Brazilian version of the Frontal Assessment Battery (FAB).
Preliminary data on administration to healthy elderly. Dement Neuropsychol.

[41] Dubois B, Slachevsky A, Litvan I, Pillon B (2000). The FAB. A frontal assessment battery at bedside. Neurology.

[42] Hurt CS, Burns A, Brown RG, Barrowclough C (2010). Perceptions of subjective memory complaint in older adults: the
Illness Perception Questionnaire- Memory (IPQ-M). Int Psychogeriatr.

[43] Smith G, Della Sala S, Logie RH, Maylor EA (2000). Prospective and retrospective memory in normal ageing and
dementia: a questionnaire study. Memory.

[44] Crook TH 3rd, Feher EP, Larrabee GJ (1992). Assessment of memory complaint in age-associated memory
impairment: the MAC-Q. Int Psychogeriatr.

[45] Rami L, Bosch B, Sanchez-Valle R, Molinuevo JL (2010). The memory alteration test (M@T) discriminates between subjective
memory complaints, mild cognitive impairment and Alzheimer's
disease. Arch Gerontol Geriatr.

[46] Vale FAC, Balieiro-Júnior AP, Silva-Filho JH (2008). P3-186: Subjective memory complaint in Alzheimer's
disease. Alzheimers Dement.

[47] Silva-Filho JH, Balieiro-Júnior AP, Vale FAC (2007). The Questionnaire of Subjective Memory Complaints in Manaus -
preliminary results. Dement Neuropsychol.

[48] Balieiro-Júnior AP, Vale FAC, Silva-Filho JH (2011). MCQ - An Instrument to Assess Memory Complaints. Dement Neuropsychol.

[49] Kashiwa Y, Kitabayashi Y, Narumoto J, Nakamura K, Ueda H, Fukui K (2005). Anosognosia in Alzheimer's disease: Association with patient
characteristics, psychiatric symptoms and cognitive deficits. Psychiatry Clin Neurosci.

[50] Hurt CS, Burns A, Brown RG, Barrowclough C (2010). Perceptions of subjective memory complaint in older adults: the
Illness Perception Questionnaire - Memory (IPQ-M). Int Psychogeriatr.

